# Structure-Preserving Point Cloud Completion with Symmetry-Guided Progressive Refinement

**DOI:** 10.3390/s26113536

**Published:** 2026-06-03

**Authors:** Shuanfeng Zhao, Yixin Niu

**Affiliations:** School of Mechanical Engineering, Xi’an University of Science and Technology, Xi’an 710054, China; 23205108049@stu.xust.edu.cn

**Keywords:** point cloud completion, symmetry-guided completion, graph neural network, transformer, progressive refinement, 3D reconstruction

## Abstract

Point cloud completion from partial observations remains challenging due to the trade-off between preserving global structural consistency and recovering fine-grained local details, especially under severe incompleteness. We propose a symmetry-guided progressive refinement network to address this problem by learning flexible structural correspondences and progressively refining incomplete shapes. First, a Symmetry Graph Inference Network (SymGraphNet) constructs a feature-space graph over sampled keypoints and predicts symmetry-guided structural counterparts for robust coarse shape recovery, without explicitly estimating a rigid symmetry plane or axis. Second, a confidence-weighted Cross-Aware Decoder adaptively fuses partial-observation features and symmetry-guided features to balance visible-region fidelity and missing-region completion. Third, a multi-stage residual refinement strategy progressively improves geometric fidelity, local continuity, and point distribution uniformity. Experiments on PCN, MVP, and KITTI datasets demonstrate consistent improvements over representative state-of-the-art methods under both synthetic and real-world incomplete point cloud settings.

## 1. Introduction

Point cloud completion is a fundamental challenge in 3D vision with broad applications in autonomous driving, robotics, and digital reconstruction [1,2]. However, real-world point clouds often suffer from severe incompleteness caused by occlusions, limited viewpoints, and sensor sparsity [3]. Such incompleteness can degrade downstream tasks such as recognition, pose estimation, and 3D reconstruction.

The key difficulty lies in balancing two conflicting objectives: maintaining global structural consistency while recovering fine-grained local details. Existing methods tend to emphasize one aspect at the expense of the other. Structure-oriented approaches, such as voxel- or grid-based networks [4], can preserve coarse geometry but may suffer from quantization artifacts and distorted structures. Point-based generative models, including coarse-to-fine decoders, tree-structured decoders, fractal networks, and Transformer-based methods [5,6,7,8,9], directly operate on point sets and improve local detail recovery, but they may still produce incomplete local regions or globally inconsistent shapes under severe missing observations. Geometry-guided and symmetry-guided methods [10,11] further introduce structural priors to improve shape consistency. Nevertheless, methods that rely on explicit or rigid symmetry assumptions may be less flexible when objects contain imperfect, local, or ambiguous symmetry.

To address these challenges, we propose a *symmetry-guided progressive refinement* framework for point cloud completion. Instead of explicitly estimating a symmetry plane or axis, the proposed method learns flexible symmetry-guided structural correspondences in the feature-space and progressively refines the coarse structure by fusing partial-observation cues and symmetry-guided cues. The main contributions are summarized as follows:

- **SymGraphNet**: We propose a graph–Transformer hybrid module for symmetry-guided structural correspondence learning. It constructs a feature-space *k*-NN graph among sampled keypoints and predicts residual counterpart points through graph attention message passing and Transformer-based contextual refinement, avoiding the need for explicit symmetry plane or symmetry-axis estimation.
- **Confidence-weighted Cross-Aware Decoder**: We design a dual cross-attention decoder that adaptively fuses partial-observation features and symmetry-guided features. A token-wise confidence gate is introduced to balance visible-region fidelity and missing-region completion during refinement.
- **Progressive refinement strategy**: We develop a multi-stage residual decoding scheme that gradually refines the rough symmetry-guided coarse structure into a denser and more geometrically consistent point cloud, improving local continuity and point distribution uniformity.

Extensive experiments on PCN [5], MVP [12], and KITTI [3] datasets demonstrate that the proposed approach achieves consistent improvements over representative state-of-the-art methods under both synthetic and real-world incomplete point cloud settings.

## 2. Related Work

Deep learning-based methods for 3D point cloud completion can be broadly categorized into voxel/mesh-based methods, point-based generative methods, and geometry-guided methods. These methods differ in their shape representations, decoding mechanisms, and ability to preserve structural consistency under severe incompleteness.

**Voxel/mesh-based methods.** Voxel- and mesh-based methods convert irregular point sets into structured representations, such as voxel grids or meshes, so that convolutional operations can be applied for feature extraction and shape reconstruction. GRNet [4] introduced a gridding residual representation to recover dense point clouds from incomplete observations and showed improved contour reconstruction. However, voxelization inevitably introduces quantization artifacts and increases memory consumption, which limits the reconstruction of thin structures and fine geometric details, especially at high resolutions.

**Point-based generative methods.** Point-based methods operate directly on unordered point sets and avoid the resolution limitation of voxelized representations. PCN [5] adopted a coarse-to-fine decoding strategy and became a representative baseline for point cloud completion. TopNet [6] generated complete point clouds through a tree-structured decoder, while PFNet [7] exploited multi-scale fractal features to recover local details. SnowflakeNet [8] further improved fine-grained reconstruction by progressively expanding points through snowflake point deconvolution. Transformer-based methods such as PointTr [9] enhanced global feature modeling by capturing long-range dependencies among point tokens. More recent methods, including AnchorFormer [13] and ODG [14], further improved completion quality through discriminative node selection and dictionary-guided shape representation. Nevertheless, these methods may still produce local holes, sparse regions, or globally inconsistent structures when the input point cloud is severely incomplete.

**Geometry-guided and symmetry-guided methods.** Geometry-guided methods introduce structural priors to improve shape plausibility and consistency. GTNet [11] learns geometric transformations for point cloud completion, showing that transformation-based priors can help recover missing structures. Symmetry has also been explored as an effective prior because many man-made objects contain repeated, mirrored, or structurally corresponding parts. SymmCompletion [10] is a representative symmetry-guided method that estimates point-wise local symmetry transformations and uses symmetry-derived geometric cues to guide point cloud refinement. These methods demonstrate the value of geometric priors for improving reconstruction consistency. However, transformation-based symmetry guidance may still be sensitive to imperfect, local, or ambiguous symmetry, especially when the observed partial input provides insufficient evidence for reliable counterpart estimation.

**Semantic scene completion and structural reasoning.** Beyond object-level point cloud completion, semantic scene completion aims to jointly infer the geometry and semantic labels of occluded 3D scenes from incomplete observations. Recent methods have explored structural reasoning from incomplete scene representations. CVSformer [15] introduces cross-view synthesis and a Transformer-based architecture to model cross-view relationships for semantic scene completion. Voxel Proposal Network [16] further improves scene completion by generating voxel proposals and using multi-frame knowledge distillation to enhance semantic and geometric prediction. These studies demonstrate the importance of exploiting structural relationships from incomplete observations at the scene level. Different from semantic scene completion, which focuses on voxel occupancy and semantic label prediction in large-scale scenes, our work addresses object-level point cloud completion and focuses on recovering dense geometric point sets from partial observations. Nevertheless, both research lines share a common motivation: leveraging structural cues from incomplete 3D observations to improve completion quality. Recent editorial discussions on intelligent point cloud processing, sensing, and comprehension [17] also highlight the broader importance of robust 3D perception, completion, and understanding.

**Comparison with SymmCompletion.** Among existing geometry-guided approaches, SymmCompletion [10] is the most closely related to our work, as both methods exploit symmetry cues to improve structural consistency in point cloud completion. SymmCompletion consists of a Local Symmetry Transformation Network (LSTNet) and a Symmetry-Guidance Transformer (SGFormer). LSTNet estimates point-wise local symmetry transformations to map key geometries from partial inputs into missing regions, thereby generating geometry-aligned partial-missing pairs and initial point clouds. SGFormer then uses these symmetry-derived geometric cues as explicit guidance to refine the initial point clouds.

In contrast, our method differs from SymmCompletion in two main aspects. First, for coarse prior construction, we do not directly rely on local symmetry transformation alone. Instead, we introduce a Symmetry Graph Inference Network (SymGraphNet) to construct a feature-space *k*-NN graph among sampled keypoints, aggregate structural relations through graph attention, and refine contextual embeddings with a Transformer before predicting residual counterpart displacements. Therefore, the generated coarse prior is not only symmetry-guided but also explicitly structure-aware. Second, for refinement, our Cross-Aware Decoder introduces a token-wise confidence gate to adaptively balance partial-observation cues and symmetry-guided cues. This allows the model to dynamically adjust the contribution of the two feature streams according to local contextual reliability during progressive refinement.

To further clarify the distinction between SymmCompletion and the proposed framework, their main methodological differences are summarized in Table 1.

In summary, voxel-based methods are constrained by discretization resolution, point-based generative methods may struggle to maintain structural consistency under extreme sparsity, and existing geometry-guided methods often depend on transformation-based or explicit symmetry guidance. These limitations motivate the development of a symmetry-guided progressive refinement framework that learns flexible structural correspondences and adaptively fuses partial-observation and symmetry-guided cues.

## 3. Method

**Figure 1:**
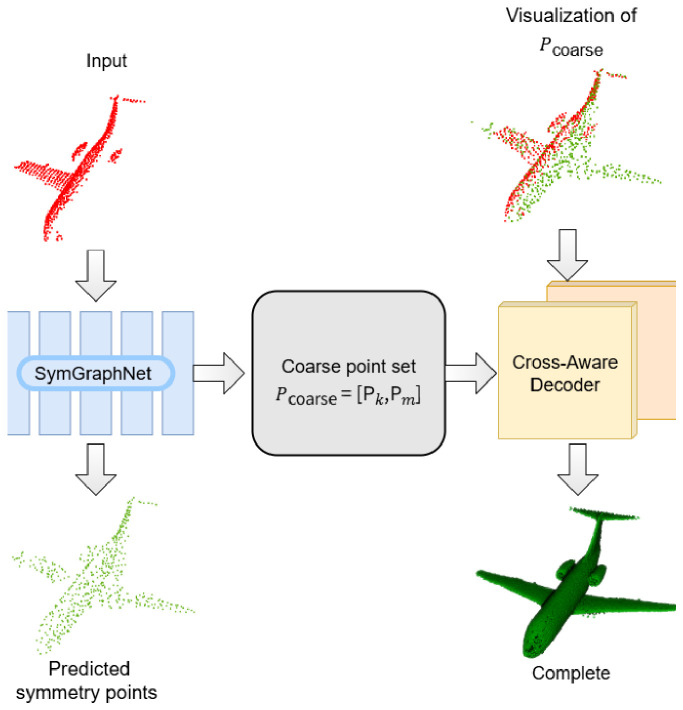
Overall framework of the proposed symmetry-guided completion network.

Our goal is to complete highly incomplete point clouds by jointly preserving global structural consistency and local geometric fidelity. To this end, we design a *symmetry-guided progressive refinement* framework that integrates symmetry-guided structural correspondence learning, cross-aware feature fusion, and multi-stage residual refinement. The overall pipeline is illustrated in Figure 1.

**Overall architecture:** The proposed framework consists of two major components: a Symmetry Graph Inference Network (SymGraphNet) and a Cross-Aware Decoder. SymGraphNet first infers symmetry-guided structural counterparts from the partial input and generates a structurally plausible coarse prior. The Cross-Aware Decoder then progressively refines this coarse prior by adaptively fusing partial-observation cues and symmetry-guided cues through confidence-weighted dual cross-attention. These two modules are connected in a coarse-to-fine manner, allowing the network to first recover the global structure and then progressively improve local geometry and point distribution.

**Figure 2:**
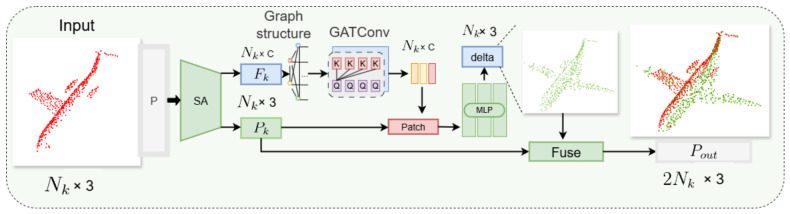
Pipeline of the Symmetry Graph Inference Network (SymGraphNet). Given an incomplete input point set, a set abstraction (SA) module samples keypoints $P_{k}\in R^{N_{k}\times 3}$ and extracts local descriptors $F_{k}\in R^{N_{k}\times C}$. A *k*-NN graph is constructed in the feature-space and processed by GATConv to aggregate structural relations, followed by a patch-level Transformer to refine contextual embeddings. The refined feature is mapped by an MLP to regress residual displacements $\Delta P\in R^{N_{k}\times 3}$, producing symmetry-guided structural counterparts $P_{m}=P_{k}+\Delta P$. Finally, the keypoints and their counterpart points are fused to form the coarse output $P_{\mathrm{out}}\in R^{2N_{k}\times 3}$.

**Symmetry Graph Inference Network (SymGraphNet):** Given a partial point cloud $P\in R^{N\times 3}$, SymGraphNet aims to generate a coarse structure by predicting symmetry-guided structural counterparts for a subset of sampled keypoints. The detailed pipeline of SymGraphNet is shown in Figure 2.

As illustrated in Figure 2, SymGraphNet follows a bottom-up structural inference routine. The incomplete input point set is first processed by a set abstraction (SA) module to obtain a compact set of keypoints and their local descriptors. These keypoint descriptors are then used to construct a feature-space *k*-NN graph, where each node corresponds to a sampled keypoint and each edge connects structurally related keypoints according to feature similarity. GATConv is applied on this graph to aggregate local structural relations with attention weights. The resulting graph-enhanced features are further processed by a patch-level Transformer to capture long-range dependencies among keypoints. Finally, the refined contextual features are combined with the keypoint coordinates and mapped by shared MLPs to predict residual displacements. The displaced points are treated as symmetry-guided structural counterparts, and they are concatenated with the original keypoints to form the coarse output.

Specifically, we first adopt a PointNet++-style set abstraction module with farthest point sampling (FPS) and *k*-nearest-neighbor grouping [18] to sample $N_{k}$ keypoints and aggregate local neighborhoods, producing keypoint coordinates $P_{k}\in R^{N_{k}\times 3}$ and local descriptors $F_{k}\in R^{N_{k}\times C}$. In our implementation, this module is implemented as PointNet_SA_Module_KNN. It samples $N_{k}=512$ keypoints using FPS and groups 16 nearest neighbors around each sampled keypoint for local feature aggregation. The grouped local features are then processed by shared MLP layers and max pooling to obtain local descriptors.

To model structural correlations among keypoints beyond local Euclidean neighborhoods, we construct a *k*-NN graph *in the feature-space* using $F_{k}$, rather than directly using Euclidean coordinates. In our implementation, k=16. This feature-space graph allows keypoints with similar structural roles to exchange information even when they are spatially distant in the partial observation.

Graph attention message passing [19] is then performed on the feature-space graph to propagate structural cues along the learned edges. The GAT module provides topology-aware local structural aggregation, where the contribution of each neighboring keypoint is adaptively weighted by attention. The graph-updated keypoint features are further refined by a Transformer encoder [20] to capture long-range dependencies among all keypoint tokens, yielding contextual embeddings $F_{\mathrm{trans}}\in R^{N_{k}\times C}$. In this design, GAT and Transformer play complementary roles: GAT focuses on feature-space graph neighborhoods, whereas the Transformer models global token-level context.

Instead of explicitly estimating a symmetry plane or axis, we learn a point-wise symmetry-guided structural code that summarizes both local geometry and contextual information. Concretely, for each keypoint, we concatenate its coordinate and contextual feature and project them with a shared MLP:

(1) $$Z_{k}=\phi \left([P_{k},F_{\mathrm{trans}}]\right),$$

where $Z_{k}\in R^{N_{k}\times C}$ denotes the symmetry-guided structural code and ϕ(·) is a learnable mapping. We then apply max pooling over keypoints to obtain a global structural descriptor $Z_{g}=\max_{k}Z_{k}$, and predict a residual displacement for each keypoint using another shared MLP:

(2) $$\Delta P=\psi \left([Z_{k},Z_{g}]\right),$$

where $\Delta P\in R^{N_{k}\times 3}$ and ψ(·) is shared across keypoints. The counterpart points are generated by residual translation:

(3) $$P_{m}=P_{k}+\Delta P.$$

The coarse set is then formed as $P_{\mathrm{coarse}}=\left[P_{k},P_{m}\right]$.

No explicit symmetry annotation, symmetry plane, or symmetry-axis supervision is used during training. Therefore, $P_{m}$ should be interpreted as symmetry-guided structural counterparts rather than strictly mirrored points. This residual counterpart generation avoids rigid reflection constraints and allows the network to learn flexible structural correspondences under imperfect, local, or partial symmetry [10,11].

**Figure 3:**
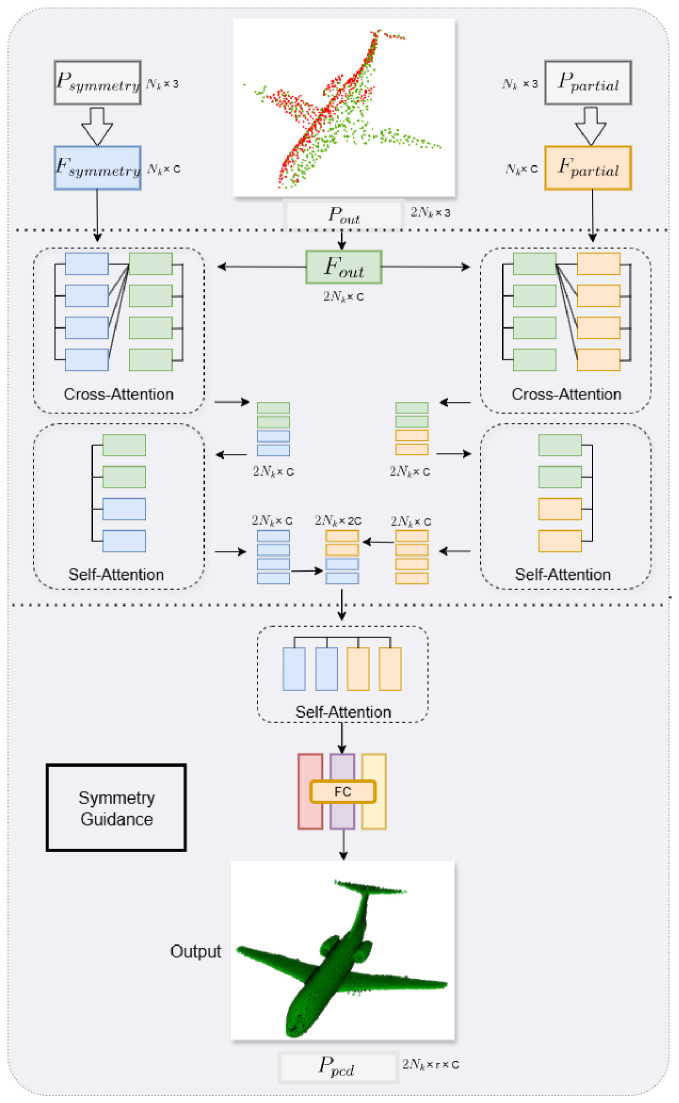
Pipeline of the Cross-Aware Decoder with confidence-weighted dual cross-attention. The decoder takes the coarse point set $P_{\mathrm{out}}\in R^{2N_{k}\times 3}$ and its token features $F_{\mathrm{out}}\in R^{2N_{k}\times C}$ as queries, and performs two cross-attention blocks to separately fuse symmetry guidance $\left(P_{\mathrm{sym}},F_{\mathrm{sym}}\right)$ and partial observations $\left(P_{\mathrm{partial}},F_{\mathrm{part}}\right)$ as keys/values. Each branch is further refined by self-attention, and a token-wise confidence gate adaptively balances the two streams before a final self-attention and an FC head regresses point offsets to generate the refined completion $P_{\mathrm{pcd}}$.

**Cross-Aware Decoder:** To refine the coarse prediction generated by SymGraphNet, we employ a two-stage Cross-Aware Decoder. The detailed structure and information flow of the decoder are shown in Figure 3.

As illustrated in Figure 3, the Cross-Aware Decoder refines the coarse output through a confidence-weighted dual-stream fusion routine. The coarse point set and its token features are used as the query stream. In parallel, the symmetry-guided points/features and the partial input points/features provide two complementary key/value streams. The symmetry-guided stream supplies structural priors for missing regions, while the partial-observation stream preserves reliable geometry from visible regions. Two cross-attention blocks are first used to separately aggregate information from these two streams. The resulting partial-aware and symmetry-aware features are then processed by self-attention to improve intra-branch consistency. A token-wise confidence gate estimates the relative reliability of the two streams and adaptively reweights them before fusion. The fused feature is further refined by self-attention and passed to an FC offset head to predict residual point displacements, producing the refined completion.

The progressive routine is implemented with two cascaded refinement stages. Let $P_{0}=P_{\mathrm{coarse}}$ denote the coarse output. The first decoder stage predicts residual offsets from $P_{0}$ and produces an intermediate reconstruction $P_{1}$. The second decoder stage takes $P_{1}$ as input and further predicts residual offsets to obtain the final dense output $P_{2}$. In this way, the decoder does not recover the complete shape in a single step, but progressively transforms a rough symmetry-guided coarse structure into a denser and more geometrically consistent point cloud.

At each refinement stage, input point features are first initialized through MLP layers and concatenated with a global feature vector. A Transformer encoder models spatial and semantic dependencies, producing enriched coarse token features. To explicitly leverage complementary information, we introduce two feature streams: symmetry-guided features ($F_{\mathrm{sym}}$) from SymGraphNet and partial-observation features ($F_{\mathrm{part}}$) from the input. After dimensional alignment, cross-attention is applied, where the coarse token features serve as queries and the two feature streams act as keys and values. Formally, letting $F\in R^{N\times d}$ denote the coarse token features, we compute two cross-attention outputs:

(4) $${\hat{F}}^{p}=\mathrm{CA}\left(F,F_{\mathrm{part}}\right),\;{\hat{F}}^{s}=\mathrm{CA}\left(F,F_{\mathrm{sym}}\right),$$

where CA(·) denotes a cross-attention block with *F* as queries and the corresponding feature stream as keys and values.

To adaptively balance observation-driven cues and symmetry-guided cues at each token, we predict a token-wise confidence gate $c\in {\left[0,1\right]}^{N\times 1}$. In implementation, *F*, ${\hat{F}}^{p}$, and ${\hat{F}}^{s}$ are first projected to the same 256-dimensional feature-space. Therefore, their concatenation has a dimension of N×(256×3)=N×768. The confidence gate takes this concatenated feature as input and outputs a scalar confidence value for each token through a two-layer MLP followed by a sigmoid function:

(5) $$c=\sigma \;\left(\mathrm{MLP}\;\left([F,{\hat{F}}^{p},{\hat{F}}^{s}]\right)\right).$$

The two branches are then weighted as:

(6) $${\tilde{F}}^{p}=c⊙{\hat{F}}^{p},\;{\tilde{F}}^{s}=\left(1-c\right)⊙{\hat{F}}^{s}.$$

The weighted features are concatenated and fused by an MLP before subsequent refinement. A self-attention layer [21] is then adopted to enhance local consistency and global coherence. Finally, fully connected layers decode residual displacements, yielding the refined output at each stage. This residual refinement formulation mitigates over-smoothing and helps preserve sharp local structures.

### Loss Function

We employ the Chamfer Distance (CD) to measure the geometric discrepancy between the generated point cloud and the complete ground truth point cloud, a metric widely used in point cloud generation and completion [22]. Let the predicted point cloud be *P* and the corresponding ground truth complete point cloud be *Q*. The Chamfer Distance is defined as:

(7) $$CD\left(P,Q\right)=\frac{1}{\left|P\right|}\sum_{p\in P}\min_{q\in Q}{∥p-q∥}_{2}^{2}+\frac{1}{\left|Q\right|}\sum_{q\in Q}\min_{p\in P}{∥p-q∥}_{2}^{2}.$$

The overall training objective is formulated as a multi-stage reconstruction loss:

(8) $$L=\sum_{i=0}^{n}CD\left(P_{i},Q\right),$$

where $P_{0}$ denotes the coarse output generated by SymGraphNet, $P_{i}$ denotes the output of the *i*-th refinement stage, *Q* is the ground truth complete point cloud, and *n* is the total number of Cross-Aware Decoder stages. In our implementation, two refinement stages are used, and the loss is applied to the coarse output, the intermediate output, and the final output.

## 4. Experiments

### 4.1. Datasets

We evaluate the proposed method on three widely used point cloud completion benchmarks: PCN, MVP, and KITTI.

**PCN** [5] is a ShapeNetCore-based benchmark containing eight object categories. Each sample consists of a partial input point cloud and a complete ground truth point cloud. The complete point clouds contain 16,384 points, while the partial inputs are generated from depth-based occlusion views. PCN is mainly used to evaluate category-level completion performance under synthetic partial observations.

**MVP** [12] provides complete shapes with 40,000 points and multiple partial views for each object. Compared with PCN, MVP contains more diverse partial observations and more complex multi-view occlusion patterns, making it suitable for evaluating robustness under challenging synthetic incompleteness.

**KITTI** [3] contains real-world LiDAR scans collected from autonomous driving scenarios. The point clouds are sparse, noisy, and incomplete, which makes KITTI suitable for evaluating the generalization ability of completion methods in practical perception scenes.

### 4.2. Evaluation Metrics

We adopt commonly used metrics for point cloud completion, including Chamfer Distance (CD), F-score, Fréchet Distance (FD), and Maximum Mean Discrepancy (MMD). For PCN, we report the L1 Chamfer Distance (CD-L1, $\times 10^{3}$) and F-score at the 1% threshold, following the standard evaluation protocol. For MVP, we report CD-L2 and F-score. For KITTI, where complete ground truth point clouds are not available in the same way as synthetic datasets, we report FD and MMD to measure distributional similarity.

CD measures the nearest-neighbor geometric discrepancy between the predicted point cloud and the ground truth. F-score evaluates the overlap between prediction and ground truth under a distance threshold by considering both precision and recall. FD and MMD measure distribution-level similarity between generated and reference point clouds. Lower CD, FD, and MMD values indicate better performance, while a higher F-score indicates better reconstruction quality.

### 4.3. Implementation and Training Details

The proposed model was implemented in PyTorch and trained on an NVIDIA GeForce RTX 4090 GPU. The software environment was Windows, PyTorch 2.1.0+cu121, CUDA 12.1, PyTorch Geometric 2.7.0, and torch-cluster 1.6.3. Unless otherwise specified, the same model architecture was used in all experiments. The network was trained using the AdamW optimizer with an initial learning rate of $2\times 10^{-4}$ and a weight decay of $5\times 10^{-4}$. We adopted a warm-up cosine learning rate scheduler. The learning rate was linearly warmed up for the first 5 epochs and then decayed from $2\times 10^{-4}$ to $1\times 10^{-5}$ following a cosine schedule. The total number of training epochs was set to 300.

For the point cloud completion network, the upsampling factors of the two progressive decoder stages were set to 2 and 8, respectively. The coarse upsampling factor was set to 2, and the original input points were not concatenated to the final output during training. The total batch size was set to 8, and the gradient update was performed once per iteration. The model selection criterion was based on the Chamfer Distance L2 (CD-L2) on the validation set.

For the PCN dataset, the training split was used for model optimization, and the official test split was used only for final evaluation. Model selection was based on the validation metric defined in the training configuration, and all reported results followed the standard benchmark protocol. During inference latency measurement, gradient computation was disabled using torch.no_grad(). All methods were evaluated with batch size 1 on the same RTX 4090 GPU to ensure a fair efficiency comparison.

### 4.4. Comparative Experiments

We compare the proposed method with representative point cloud completion methods, including PCN, GRNet, TopNet, PointTr, SnowflakeNet, AnchorFormer, ODG, and SymmCompletion. These baselines cover voxel/grid-based, point-based generative, Transformer-based, dictionary-guided, and symmetry-guided completion methods. The comparisons are conducted on PCN, MVP, and KITTI to evaluate completion performance under synthetic single-view incompleteness, synthetic multi-view occlusion, and real-world LiDAR sparsity, respectively.

**Results on PCN.** As shown in Table 2, we evaluate the proposed method on the PCN dataset and compare it with representative baselines, including PCN, GRNet, TopNet, PointTr, SnowflakeNet, AnchorFormer, ODG, and SymmCompletion. Following the standard PCN evaluation protocol, we report the L1 Chamfer Distance (CD-L1, $\times 10^{3}$; lower is better) and the F-score at the 1% threshold (higher is better).

The results in Table 2 show that the proposed method achieves the lowest average CD-L1 of 6.26 and the highest F-score of 0.852 on the PCN dataset. Compared with earlier methods such as PCN, TopNet, and GRNet, our method substantially improves both geometric accuracy and overlap quality. Compared with recent competitive methods, including SnowflakeNet, AnchorFormer, ODG, and SymmCompletion, our method remains consistently competitive and achieves the best average performance.

It should be noted that the performance margin over SymmCompletion on PCN is relatively small. Since repeated independent training of the full model and the strongest baselines requires substantial computational resources, we report single-run results following the common benchmark protocol in this revision. Therefore, the PCN comparison should be interpreted as demonstrating competitive performance against the strongest symmetry-guided baseline rather than statistically significant superiority. Multi-run mean and standard deviation results will be included in future extended evaluations when sufficient computational resources are available.

At the category level, our method obtains the best or tied-best CD-L1 on Airplane, Car, Sofa, Table, and Watercraft, and remains close to the best results on Cabinet, Chair, and Lamp. These results suggest that the proposed structure-aware symmetry-guided refinement is effective not only for highly regular categories such as Airplane and Car, but also for categories with more diverse local structures.

**Figure 4:**
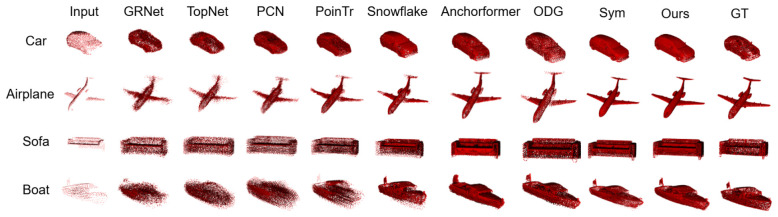
Visualization results on the PCN dataset. Each row shows one example from Car, Airplane, Sofa, and Boat. From left to right: Input, GRNet, TopNet, PCN, PointTr, SnowflakeNet, AnchorFormer, ODG, SymmCompletion (Sym), Ours, and GT.

Qualitative comparisons in Figure 4 further corroborate the quantitative trends. Across all four categories, early baselines such as GRNet, TopNet, and PCN tend to produce over-smoothed or incomplete structures under heavy missing regions. Transformer-based methods such as PointTr, AnchorFormer, and ODG improve global plausibility, but may still exhibit point sparsity, local holes, or blurred boundaries in thin structures. By contrast, the proposed method generates more compact and structurally consistent reconstructions with clearer contours and more uniform point distributions. In particular, on *Car* and *Boat*, our results better preserve the overall hull/body shape and reduce missing regions; on *Airplane*, the wings and tail are reconstructed with improved continuity; and on *Sofa*, the seat and back surfaces are more complete with fewer artifacts.

**Figure 5:**
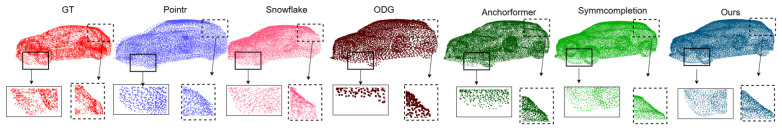
Detailed comparison on the *Car* category from the PCN dataset. Each example shows global reconstruction (**top**) and zoomed-in regions (**bottom**). Compared with PointTr, SnowflakeNet, AnchorFormer, ODG, and SymmCompletion, our method yields denser and more continuous reconstructions, closely matching the ground truth (GT).

A detailed comparison on the *Car* category is further provided in Figure 5. The zoomed-in regions show that our method produces denser and more continuous local structures, especially around boundary and surface regions, while better preserving the global vehicle shape.

**Results on MVP.** We further evaluate the proposed method on the MVP dataset, which contains more diverse partial views and more complex multi-view occlusion patterns than PCN. We compare our method with five representative baselines, including PointTr, SnowflakeNet, AnchorFormer, SymmCompletion, and ODG. Table 3 reports the category-wise CD-L2 and the overall F-score.

As shown in Table 3, the proposed method achieves the lowest average CD-L2 of 2.17 and the highest F-score of 0.874. Compared with SymmCompletion and ODG, our method improves both average CD-L2 and F-score, indicating that the proposed graph-based structural reasoning and confidence-weighted fusion are beneficial under diverse multi-view occlusion patterns.

At the category level, the proposed method achieves the best CD-L2 in seven out of 16 categories, including Chair, Table, Sofa, Lamp, Watercraft, Bench, and Bookshelf, while remaining competitive in the remaining categories. The results also show that the method performs well on both strongly structured categories and categories with weaker or more local symmetry. This suggests that SymGraphNet does not merely learn strict mirror symmetry, but captures more general symmetry-guided structural correspondences.

**Figure 6:**
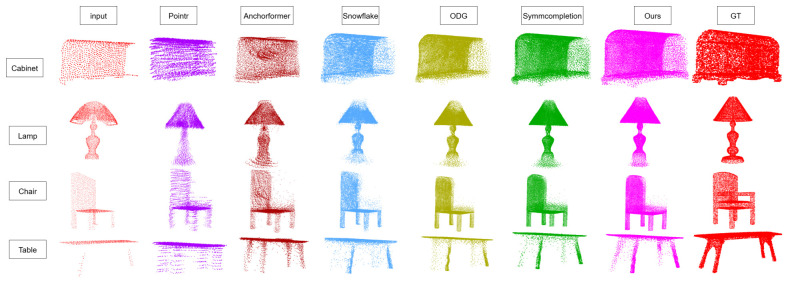
Visualization results on the MVP dataset. From left to right: input partial point cloud, representative baseline methods, our method, and ground truth. Compared with existing methods, our approach produces more complete structures, better preserves geometric continuity, and recovers finer local details under complex multi-view occlusions.

Qualitative comparisons on MVP are shown in Figure 6. Compared with the baseline methods, our method produces more complete object structures and more continuous local surfaces under complex multi-view occlusions. The improvements are particularly visible in categories with missing legs, thin structures, or incomplete planar surfaces, where the proposed progressive refinement strategy helps reduce local holes and improve point distribution uniformity.

**Results on KITTI.** To further evaluate real-world generalization, we test the proposed method on the KITTI dataset. Unlike PCN and MVP, KITTI contains sparse and noisy LiDAR scans from driving scenarios, making it more challenging for point cloud completion. Following common practice, we report Fréchet Distance (FD) and Maximum Mean Discrepancy (MMD) for quantitative evaluation.

Table 4 reports the quantitative results on KITTI. The proposed method achieves the best FD of 2.518 and the best MMD of 1.123 among the compared methods. These results indicate that the proposed framework generalizes reasonably well from synthetic training data to sparse and noisy real-world LiDAR scans.

**Figure 7:**
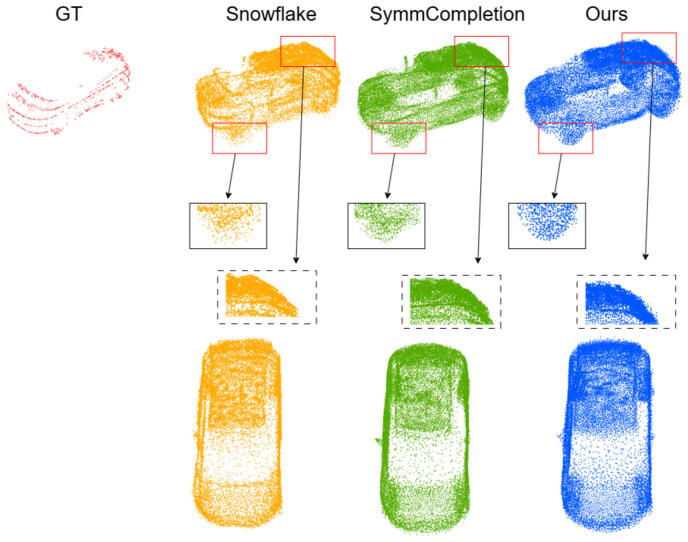
Visualization of completion results on the KITTI dataset. From left to right: reference KITTI scan (denoted as GT in the figure for visual comparison only), baseline methods (SnowflakeNet, SymmCompletion), and ours. Since KITTI does not provide complete ground truth point clouds in the same way as synthetic datasets, the leftmost point cloud is used only as a visual reference rather than complete reconstruction supervision.

The qualitative results in Figure 7 further show the visual comparison with the reference KITTI scan. Since KITTI does not provide complete ground truth shapes in the same way as synthetic datasets, this reference scan is used only for qualitative visualization. SnowflakeNet and SymmCompletion may produce missing regions or uneven point distributions around key vehicle structures such as the roof and body boundaries. In contrast, our method generates smoother and more complete vehicle shapes with more uniform point distributions. This suggests that the structure-aware coarse prior and progressive refinement strategy are helpful for real-world point cloud completion.

**Efficiency analysis.** In addition to reconstruction quality, we evaluate the inference efficiency of the proposed method. Table 5 reports the number of parameters and inference latency under the same test setting with batch size 1. Although our method has a comparable parameter count to SymmCompletion, its latency is higher. This indicates that parameter count and runtime are not strictly correlated in point cloud completion networks.

The higher latency mainly comes from three sources. First, SymGraphNet constructs an on-the-fly feature-space *k*-NN graph, which introduces additional neighbor search and indexing operations. Second, GAT-based message passing involves irregular memory access and scatter/gather operations, which are usually less GPU-friendly than dense MLP or Transformer computations. Third, the two-stage Cross-Aware Decoder performs dual cross-attention and token-wise confidence-gated fusion at each refinement stage, introducing additional computation during progressive refinement.

Nevertheless, the increased computational cost is associated with the improved structural modeling ability of the proposed method. Compared with purely point-based or Transformer-based refinement, the feature-space graph reasoning provides additional structural correspondence modeling, while the confidence-weighted decoder improves the fusion of partial-observation and symmetry-guided cues. Therefore, the proposed method provides a trade-off between reconstruction accuracy and inference efficiency.

For applications with strict real-time constraints, the current graph construction and graph attention operations may become the main computational bottleneck. Future optimization can include lightweight graph construction, cached neighborhood computation, or replacing GATConv with more efficient local aggregation operators.

## 5. Ablation Study

In this section, we conduct ablation studies to analyze the contribution of the main components in the proposed framework. Specifically, we investigate four aspects: (i) the complementarity between partial-observation features and symmetry-guided features, (ii) the roles of GAT and Transformer in SymGraphNet, (iii) the effect of the confidence-weighted fusion strategy in the Cross-Aware Decoder, and (iv) the influence of the number of refinement stages. Unless otherwise specified, all ablation experiments are conducted on the PCN dataset under the same training and evaluation settings. CD denotes the average CD-L1, and F-score is computed at the 1% threshold.

### 5.1. Effect of Partial-Observation and Symmetry-Guided Features

We first investigate the roles of partial-observation features and symmetry-guided features in point cloud completion. Taking the full model as the baseline, we progressively remove the corresponding feature branches while keeping the rest of the architecture and training strategy unchanged. This allows us to isolate and analyze the contribution of each feature stream.

The ablation settings are as follows:

1. **w/o partial_feat (only symmetry-guided branch):** In this setting, we remove the partial-observation feature branch and retain only the symmetry-guided features extracted from SymGraphNet. This experiment evaluates whether symmetry-guided cues alone can support the reconstruction when direct partial-observation features are absent.
2. **w/o symmetry_feat (only partial-observation branch):** Here we preserve the partial-observation feature branch while removing the symmetry-guided branch. This setting is used to analyze the model’s performance without explicit symmetry-guided structural information.
3. **w/o both (coarse only):** In this setting, both partial-observation and symmetry-guided feature branches are removed, and the network relies only on coarse encoded features for completion. This serves as a lower-bound reference and highlights the overall contribution of the two feature streams.

**Figure 8:**
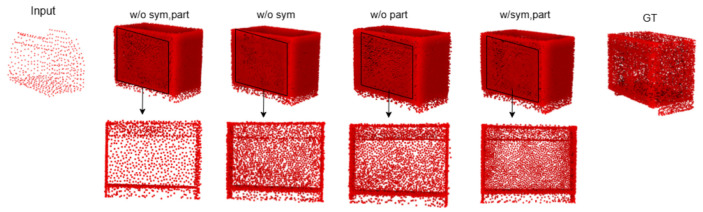
Qualitative ablation results on the *Table* category. Top row shows the overall completed shapes and the bottom row provides zoomed-in views of the highlighted regions (black boxes) for a clearer comparison of surface continuity and point uniformity. From left to right: input partial point cloud, w/o sym&part, w/o sym, w/o part, full model (w/sym&part), and ground truth (GT). Removing both branches yields the most blurred and incomplete surfaces; using only one branch improves either global integrity or local detail, while the full model best restores planar structures with denser, more uniform points and sharper boundaries.

As shown in Table 6, removing both feature branches yields the weakest performance, with a CD of 6.59 and an F-score of 0.830. Keeping only the symmetry-guided branch or only the partial-observation branch improves the results, indicating that both feature streams provide useful information for completion. However, either single branch remains inferior to the full model. When both partial-observation and symmetry-guided features are enabled, the model achieves the best performance, with a CD of 6.26 and an F-score of 0.852. This demonstrates that the two feature streams are complementary: partial-observation features help preserve reliable visible geometry, while symmetry-guided features provide structural cues for missing regions. The qualitative results in Figure 8 further confirm that the full model produces more continuous surfaces and more uniform point distributions.

### 5.2. Effect of Structural Modeling in SymGraphNet

We further verify the structural modeling components in SymGraphNet. Although GAT and Transformer are both attention-based modules, they operate on different relation spaces. GAT performs topology-aware message passing on the feature-space *k*-NN graph, where each keypoint aggregates information from structurally related neighbors. In contrast, the Transformer refines the graph-enhanced keypoint features by modeling long-range dependencies among all keypoint tokens. Therefore, these two modules are designed to be complementary rather than redundant.

As shown in Table 7, removing either GAT or Transformer degrades the reconstruction performance. The model without GAT obtains a CD of 6.54 and an F-score of 0.834, indicating that feature-space graph message passing is beneficial for structural relation aggregation. Removing the Transformer further decreases the performance to 6.58/0.832, suggesting that long-range contextual refinement among keypoints is also necessary. When both GAT and Transformer are removed, the performance drops to 6.78/0.828, showing the weakest structural modeling ability. The full model achieves the best result of 6.26/0.852, confirming that GAT and Transformer play complementary roles in SymGraphNet.

### 5.3. Effect of Confidence-Weighted Fusion

We then evaluate the fusion strategy in the Cross-Aware Decoder. The decoder is designed to fuse partial-observation features and symmetry-guided features through dual cross-attention and a token-wise confidence gate. To verify the necessity of this design, we compare the full decoder with two variants: simple feature concatenation and dual cross-attention without the confidence gate.

Table 8 shows that simple feature concatenation gives the weakest result, with a CD of 6.55 and an F-score of 0.835. This indicates that directly concatenating the two feature streams is insufficient to fully exploit the complementary information from partial observations and symmetry-guided cues. Introducing dual cross-attention improves the result to 6.48/0.839, demonstrating that separate cross-stream interaction is useful for feature fusion. The full decoder with the confidence gate achieves the best performance of 6.26/0.852. This confirms that token-wise reliability estimation helps the decoder adaptively balance observation-driven cues and symmetry-guided cues during refinement.

### 5.4. Effect of the Number of Refinement Stages

Finally, we analyze the influence of the number of refinement stages. The proposed decoder adopts a two-stage progressive refinement strategy. To verify this design choice, we compare one-stage, two-stage, and three-stage variants under the same output resolution setting.

As shown in Table 9, using only one refinement stage leads to inferior reconstruction performance, with a CD of 6.40 and an F-score of 0.843. This suggests that a single residual refinement step is not sufficient to fully recover fine-grained geometric details from the coarse prediction. Increasing the number of stages from one to two clearly improves the performance to 6.26/0.852, demonstrating the effectiveness of progressive refinement. When a third refinement stage is added, the CD is only slightly improved from 6.26 to 6.25, while the F-score remains unchanged and the latency increases from 18.55 ms to 24.80 ms. Therefore, the two-stage setting is adopted in the final model because it provides the best trade-off between reconstruction quality and inference efficiency.

## 6. Conclusions

This paper presented a symmetry-guided progressive refinement framework for point cloud completion under severe incompleteness. The proposed method integrates three key components: (i) a Symmetry Graph Inference Network (SymGraphNet) that learns symmetry-guided structural counterparts through feature-space graph reasoning and Transformer-based contextual refinement, (ii) a confidence-weighted Cross-Aware Decoder that adaptively fuses partial-observation cues and symmetry-guided cues, and (iii) a multi-stage residual refinement strategy that progressively improves geometric fidelity, local continuity, and point distribution uniformity. Extensive experiments on PCN, MVP, and KITTI demonstrate that the proposed method achieves consistent improvements over representative baselines in both quantitative metrics and qualitative reconstruction results.

Compared with conventional point-based decoders such as PCN and TopNet, the proposed method better preserves global structural consistency under severe incompleteness. Compared with Transformer-based methods such as PointTr and AnchorFormer, SymGraphNet further introduces feature-space graph reasoning to explicitly model structural relations among keypoints. Compared with symmetry-guided methods such as SymmCompletion, our method does not explicitly estimate a rigid symmetry plane or axis. Instead, it learns flexible symmetry-guided structural correspondences and progressively refines the coarse structure by adaptively balancing partial-observation and symmetry-guided features. The ablation studies further confirm the complementary roles of GAT and Transformer in SymGraphNet, the effectiveness of the confidence-weighted fusion strategy, and the favorable trade-off achieved by the two-stage refinement design.

Despite these advantages, several limitations remain. First, the symmetry-guided prior may be less reliable for objects with weak, ambiguous, or multiple local symmetries, where the predicted structural counterparts may provide biased guidance. Second, SymGraphNet relies on feature-space *k*-NN graph construction and GAT-based message passing, which introduce irregular memory access and additional computational overhead compared with purely dense MLP or Transformer pipelines. Third, the current design mainly models a dominant structural correspondence pattern at the instance level, and its robustness in strongly asymmetric, highly cluttered, or extremely sparse real-world scenes still requires further investigation. In future work, we will explore lightweight or cached graph construction to reduce runtime cost, extend the symmetry-guided modeling to multi-hypothesis or local structural correspondence learning, and incorporate more general geometric priors such as part-level structural constraints to improve robustness in complex real-world scenarios.

## Figures and Tables

**Table 1:** Methodological comparison between SymmCompletion and the proposed framework.

Aspect	SymmCompletion	Ours
Symmetry modeling strategy	Estimates point-wise local symmetry transformations to map key geometries from partial inputs into missing regions.	Learns symmetry-guided structural counterparts through feature-space graph reasoning and residual displacement prediction.
Coarse prior construction	Generates geometry-aligned partial-missing pairs and initial point clouds based on local symmetry transformation.	Constructs a structure-aware coarse prior by combining feature-space *k*-NN graph construction, GAT-based structural aggregation, Transformer refinement, and residual counterpart prediction.
Structural relation modeling	Focuses on local symmetry transformation and symmetry-guided refinement.	Explicitly models keypoint-level structural relations through feature-space graph attention before counterpart generation.
Refinement mechanism	Uses SGFormer to exploit symmetry-derived geometric cues for refining initial point clouds.	Uses a confidence-weighted Cross-Aware Decoder to progressively fuse partial-observation features and symmetry-guided features.
Reliability-aware fusion	Uses symmetry-derived geometric cues as explicit guidance during refinement.	Introduces a token-wise confidence gate to adaptively reweight partial-observation cues and symmetry-guided cues according to contextual reliability.
Design emphasis	High-fidelity and high-consistency completion with local symmetry transformation and symmetry-guided refinement.	Structure-aware symmetry-guided coarse prior construction and confidence-aware progressive refinement.

**Table 2:** Quantitative results on the PCN dataset (CD-L1 $\times 10^{3}$, ↓ lower is better; F-score at 1%, ↑ higher is better). Best results are in bold.

Method	Airplane	Cabinet	Car	Chair	Lamp	Sofa	Table	Watercraft	Avg. CD	F-Score
PCN [5]	6.73	12.51	10.11	12.94	12.70	13.48	10.44	9.88	11.06	0.559
GRNet [4]	7.34	12.64	10.31	13.25	13.96	14.37	11.22	10.19	11.66	0.529
PointTr [9]	5.14	10.92	9.06	9.91	7.85	11.74	7.87	7.74	8.77	0.698
TopNet [6]	6.77	12.74	10.92	13.32	11.66	14.32	10.65	10.44	11.35	0.513
SnowflakeNet [8]	4.81	10.33	8.91	8.56	6.67	10.53	7.58	6.59	8.00	0.741
AnchorFormer [13]	3.89	9.13	7.95	7.37	5.75	8.74	6.27	6.06	6.89	0.817
ODG [14]	3.81	8.82	7.63	6.94	5.26	8.49	5.87	5.78	6.57	0.826
SymmCompletion [10]	**3.54**	**8.48**	**7.33**	**6.62**	**4.95**	8.25	5.65	5.53	6.29	0.851
**Ours**	**3.54**	8.49	**7.33**	6.64	4.99	**8.20**	**5.64**	**5.26**	**6.26**	**0.852**

**Table 3:** Quantitative results on the MVP dataset (CD-L2 ↓; F-score ↑). Methods are ordered by overall F-score from low to high. Best results are in bold.

Category	PointTr	SnowflakeNet	AnchorFormer	SymmCompletion	ODG	Ours
Chair	3.79	4.01	2.98	2.72	2.82	**2.57**
Table	4.03	4.08	2.94	2.80	3.21	**2.78**
Sofa	3.69	3.71	3.06	3.22	3.08	**2.92**
Cabinet	3.30	3.29	3.17	3.36	**3.10**	3.26
Lamp	4.92	5.73	3.53	2.75	3.71	**2.67**
Car	2.70	2.28	2.24	2.14	**1.95**	2.05
Airplane	1.32	1.19	0.95	**0.73**	0.79	0.76
Watercraft	2.45	3.08	2.07	2.41	2.22	**1.96**
Bed	6.52	7.18	**5.52**	5.95	6.23	5.70
Bench	2.47	2.96	2.07	1.81	2.09	**1.67**
Bookshelf	3.92	4.57	3.16	2.93	3.21	**2.83**
Bus	2.06	1.79	1.72	2.09	**1.68**	1.90
Guitar	0.67	0.41	0.38	0.32	**0.31**	**0.31**
Motorbike	2.01	1.81	1.60	1.28	**1.24**	1.28
Pistol	1.62	1.77	1.36	**1.09**	1.18	1.21
Skateboard	1.14	3.32	1.05	**0.60**	1.71	0.79
Avg. CD	2.91	3.20	2.36	2.26	2.41	**2.17**
F-score	0.790	0.827	0.830	0.862	0.866	**0.874**

**Table 4:** KITTI results: Fréchet Distance (FD, ↓) and Maximum Mean Discrepancy (MMD, ↓). Best is in bold.

Method	FD (↓)	MMD (↓)
SnowflakeNet [8]	2.733	1.143
SymmCompletion [10]	2.858	1.1248
**Ours**	**2.518**	**1.123**

**Table 5:** Inference efficiency comparison under the same setting (batch size 1). Params denotes the number of model parameters (M) and Latency denotes the average runtime per sample (ms).

Method	Params (M)	Latency (ms)
SymmCompletion [10]	13.28	10.58
SnowflakeNet [8]	15.92	18.94
Ours	13.04	18.55

**Table 6:** Ablation study on the contributions of partial-observation and symmetry-guided features. CD denotes the average CD-L1 on PCN. Best results are in bold.

Partial_Feat	Symmetry_Feat	CD (↓)	F-Score (↑)
×	×	6.59	0.830
×	✓	6.56	0.835
✓	×	6.52	0.836
✓	✓	**6.26**	**0.852**

**Table 7:** Ablation study on the complementary roles of GAT and Transformer in SymGraphNet. CD denotes the average CD-L1 on PCN. Best results are in bold.

Variant	CD (↓)	F-Score (↑)
w/o GAT	6.54	0.834
w/o Transformer	6.58	0.832
w/o GAT and Transformer	6.78	0.828
Full model	**6.26**	**0.852**

**Table 8:** Ablation study on the fusion strategy in the Cross-Aware Decoder. CD denotes the average CD-L1 on PCN. Best results are in bold.

Variant	CD (↓)	F-Score (↑)
Simple feature concatenation	6.55	0.835
Dual cross-attention w/o confidence gate	6.48	0.839
Full decoder with confidence gate	**6.26**	**0.852**

**Table 9:** Ablation study on the number of refinement stages. CD denotes the average CD-L1 on PCN. Best results are in bold.

Number of Stages	CD (↓)	F-Score (↑)	Latency (ms)
1 stage	6.40	0.843	12.30
2 stages	6.26	**0.852**	18.55
3 stages	**6.25**	**0.852**	24.80

## Data Availability

The datasets used in this study are publicly available from the corresponding benchmark sources. The implementation code, configuration files, trained model weights, and evaluation scripts will be made publicly available upon acceptance. Additional data supporting the findings of this study are available from the corresponding author upon reasonable request.
